# Supplementary material for: Riboflavin metabolism shapes FSP1-driven ferroptosis resistance
Source: Nat Cell Biol. 2026 Mar 13;28(4):696–706. doi: 10.1038/s41556-025-01856-x (PMC13086581; doi:10.1038/s41556-025-01856-x)
Supplement: Supplementary file 1 — Reporting Summary [file 41556_2025_1856_MOESM1_ESM.pdf]

Corresponding author(s): José Pedro Friedmann Angeli
Last updated by author(s): Nov 1, 2025

Reporting Summary

Nature Portfolio wishes to improve the reproducibility of the work that we publish. This form provides structure for consistency and transparency in reporting. For further information on Nature Portfolio policies, see our Editorial Policies and the Editorial Policy Checklist.

Statistics

For all statistical analyses, confirm that the following items are present in the figure legend, table legend, main text, or Methods section.

n/a	Confirmed	
☐	☒	The exact sample size (*n*) for each experimental group/condition, given as a discrete number and unit of measurement
☐	☒	A statement on whether measurements were taken from distinct samples or whether the same sample was measured repeatedly
☐	☒	The statistical test(s) used AND whether they are one- or two-sided<br>*Only common tests should be described solely by name; describe more complex techniques in the Methods section.*
☒	☐	A description of all covariates tested
☐	☒	A description of any assumptions or corrections, such as tests of normality and adjustment for multiple comparisons
☐	☒	A full description of the statistical parameters including central tendency (e.g. means) or other basic estimates (e.g. regression coefficient) AND variation (e.g. standard deviation) or associated estimates of uncertainty (e.g. confidence intervals)
☐	☒	For null hypothesis testing, the test statistic (e.g. *F*, *t*, *r*) with confidence intervals, effect sizes, degrees of freedom and *P* value noted<br>*Give P values as exact values whenever suitable.*
☒	☐	For Bayesian analysis, information on the choice of priors and Markov chain Monte Carlo settings
☒	☐	For hierarchical and complex designs, identification of the appropriate level for tests and full reporting of outcomes
☒	☐	Estimates of effect sizes (e.g. Cohen's *d*, Pearson's *r*), indicating how they were calculated

Our web collection on statistics for biologists contains articles on many of the points above.

Software and code

Policy information about availability of computer code

Data collection	Spark Control v3.2, BD FACSDiva Software v6.1.3, Amersham ImageQuant 800 v2.0.0, SnapGene v8.0.1, Incucyte v2023A Rev2, TraceFinder v5.1, Realplex v2.2.2, CFX Maestro v2.0, Schrödinger v.2024.2, AmberTools23, Gaussian 09, GROMACS v2024.2 and NanoDrop One v2.8.0.25
Data analysis	GraphPad Prism v10.4.1, Excel v24.11, MAGeCK v0.5.9, R v4.2.0, RStudio v2022.02.0, Python v3.10.2, LPPtiger2.0, Skyline v24.1.0.199, MetaboAnalyst v6.0 (online platform: https://www.metaboanalyst.ca), Spectronaut v19.1.240724.62635, ImageJ 1.54m and FlowJo v10.10.0

For manuscripts utilizing custom algorithms or software that are central to the research but not yet described in published literature, software must be made available to editors and reviewers. We strongly encourage code deposition in a community repository (e.g. GitHub). See the Nature Portfolio guidelines for submitting code & software for further information.

Data

Policy information about availability of data

All manuscripts must include a data availability statement. This statement should provide the following information, where applicable:

- Accession codes, unique identifiers, or web links for publicly available datasets
- A description of any restrictions on data availability
- For clinical datasets or third party data, please ensure that the statement adheres to our policy

Mass spectrometry data have been deposited in ProteomeXchange with the primary accession code PXD061038 (https://www.ebi.ac.uk/pride/archive/projects/PXD061038). All other data supporting the findings of this study are available from the corresponding author on reasonable request.

Research involving human participants, their data, or biological material

Policy information about studies with human participants or human data. See also policy information about sex, gender (identity/presentation), and sexual orientation and race, ethnicity and racism.

Reporting on sex and gender	*Use the terms sex (biological attribute) and gender (shaped by social and cultural circumstances) carefully in order to avoid confusing both terms. Indicate if findings apply to only one sex or gender; describe whether sex and gender were considered in study design; whether sex and/or gender was determined based on self-reporting or assigned and methods used. Provide in the source data disaggregated sex and gender data, where this information has been collected, and if consent has been obtained for sharing of individual-level data; provide overall numbers in this Reporting Summary. Please state if this information has not been collected. Report sex- and gender-based analyses where performed, justify reasons for lack of sex- and gender-based analysis.*
Reporting on race, ethnicity, or other socially relevant groupings	*Please specify the socially constructed or socially relevant categorization variable(s) used in your manuscript and explain why they were used. Please note that such variables should not be used as proxies for other socially constructed/relevant variables (for example, race or ethnicity should not be used as a proxy for socioeconomic status). Provide clear definitions of the relevant terms used, how they were provided (by the participants/respondents, the researchers, or third parties), and the method(s) used to classify people into the different categories (e.g. self-report, census or administrative data, social media data, etc.) Please provide details about how you controlled for confounding variables in your analyses.*
Population characteristics	*Describe the covariate-relevant population characteristics of the human research participants (e.g. age, genotypic information, past and current diagnosis and treatment categories). If you filled out the behavioural & social sciences study design questions and have nothing to add here, write "See above."*
Recruitment	*Describe how participants were recruited. Outline any potential self-selection bias or other biases that may be present and how these are likely to impact results.*
Ethics oversight	*Identify the organization(s) that approved the study protocol.*

Note that full information on the approval of the study protocol must also be provided in the manuscript.

Field-specific reporting

Please select the one below that is the best fit for your research. If you are not sure, read the appropriate sections before making your selection.

☒ Life sciences ☐ Behavioural & social sciences ☐ Ecological, evolutionary & environmental sciences

For a reference copy of the document with all sections, see nature.com/documents/nr-reporting-summary-flat.pdf

Life sciences study design

All studies must disclose on these points even when the disclosure is negative.

Sample size	No samples size calculation was performed. Preliminary cell viability experiments showed small variations between biological replicates, so we chose n = 3 for reproducibility.
Data exclusions	In very rare cases single values of biological triplicates were excluded from the analysis due to te cell clumps/uneven plating.
Replication	Most attempts to replicate experiments were successful, demonstrating the robustness of the results. To ensure reliable replication, all sera used were pretested for their suitability in ferroptosis research. It is well-established that variations in vitamin content, lipid composition, and selenium concentrations between serum batches profoundly impact on the outcome of ferroptosis inducing/inhibiting conditions.
Randomization	All sample handling for proteomics analysis (sample preparation and LC-MS/MS analysis) have been performed in a block randomization order.
Blinding	Blinding was not performed in this study as the experimental design required direct observation of cell phenotypes and treatment conditions were distinguishable. However, data analysis was conducted using objective, quantifiable measurements to minimize bias.

Reporting for specific materials, systems and methods

We require information from authors about some types of materials, experimental systems and methods used in many studies. Here, indicate whether each material, system or method listed is relevant to your study. If you are not sure if a list item applies to your research, read the appropriate section before selecting a response.

Materials & experimental systems

n/a	Involved in the study	
☐	☒	Antibodies
☐	☒	Eukaryotic cell lines
☒	☐	Palaeontology and archaeology
☒	☐	Animals and other organisms
☒	☐	Clinical data
☒	☐	Dual use research of concern
☒	☐	Plants

Methods

n/a	Involved in the study	
☒	☐	ChIP-seq
☐	☒	Flow cytometry
☒	☐	MRI-based neuroimaging

Antibodies

Antibodies used

GPX4 (Abcam, cat. no. ab125066 or Proteintech, cat. no. 67763-1-Ig), ACSL4 (Santa Cruz, cat. no. sc-271800), β-actin (Sigma-Aldrich, cat. no. A5441), vinculin (Santa Cruz, sc-73614), Flag-tag (Sigma-Aldrich, cat. no. F3165), FADS (Santa Cruz, cat. no. sc-376819), RFK (Santa Cruz, cat. no. sc-398830 or Abbexa cat. no. abx124688) and NQO1 (Santa Cruz, cat. no. sc-32793). The FSP1 antibody was developed in Helmholtz Zentrum München against recombinant human FSP1 protein (clone 6D8-11).

Validation

Antibodies against GPX4 (Abcam, cat. no. ab125066) were validated for Western blotting in a previous publication (PMID: 25402683). The GPX4 antibody from Proteintech (cat. no. 67763-1-Ig) was validated by the manufacturer using knockdown models (www.ptglab.com) and in our lab using knockout models.
The ACSL4 antibody (Santa Cruz, cat. no. sc-271800) was validated for Western blotting in a prior publication (PMID: 27842070).
The β-actin antibody (Sigma, cat. no. A5441) was validated as a loading control for Western blotting in a previous study (PMID: 15809369).
The vinculin antibody (Santa Cruz, cat. no. sc-73614) was validated as a loading control for Western blotting in another publication (PMID: 1478968).
The Flag-tag antibody (Sigma-Aldrich, cat. no. F3165) was validated in this study for Western Blotting, where we observed an increased signal in cells overexpressing Flag-FLAD1 compared to wild-type controls.
The FADS (Santa Cruz, cat. no. sc-376819), RFK (Santa Cruz, cat. no. sc-398830 or Abbexa, cat. no. abx124688), and NQO1 (Santa Cruz, cat. no. sc-32793) antibodies have not been fully validated according to all recommended criteria for Western blotting (e.g., validation with knockout models). For the FADS antibody, our Western blot analysis showed increased signal intensity in cells overexpressing FADS, suggesting specificity. For the RFK antibody, a clear reduction in RFK signal was observed in cells transduced with three RFK-targeting sgRNAs compared to wild-type controls, with the detected RFK band corresponding to the reported molecular weight, indicating specificity. For the NQO1 antibody, our Western blot analysis revealed a band at the expected molecular weight, and the observed expression pattern was consistent with proteomics data obtained under the same experimental conditions, as well as with previous publications (e.g., PMID: 32895367), supporting its specificity. However, these antibodies also showed non-specific bands at varying molecular weights under the tested conditions.
The FSP1 antibody was validated for Western blotting in a previous publication (PMID: 31634899).

Eukaryotic cell lines

Policy information about cell lines and Sex and Gender in Research

Cell line source(s)

Human cancer cell lines HT1080, A375, MDA-MB-231, MDA-MB-436, A549 and H460 were purchased from ATCC (cat. no. CCL-121, CRL-1619, CRM-HTB-26, HTB-130, CRM-CCL-185 and HTB-177, respectively) and PC9 was purchased from Sigma (cat. no. 90071810)

Authentication

None of the cell lines used in this study were authenticated.

Mycoplasma contamination

Cells are tested at least once a year for Mycoplasm contamination by qPCR at Eurofins Genomics.

Commonly misidentified lines
(See ICLAC register)

N/A

Plants

Seed stocks

N/A

Novel plant genotypes

N/A

Authentication

N/A

Flow Cytometry

Plots

Confirm that:

☒ The axis labels state the marker and fluorochrome used (e.g. CD4-FITC).

☒ The axis scales are clearly visible. Include numbers along axes only for bottom left plot of group (a 'group' is an analysis of identical markers).

☐ All plots are contour plots with outliers or pseudocolor plots.

☐ A numerical value for number of cells or percentage (with statistics) is provided.

Methodology

Sample preparation	50,000 cells per well were seeded on 6-well plates one day prior to the experiment. The next day, cells were treated with the following conditions (i) DMSO, (ii) RSL3 (200 nM) and (iii) RSL3 (200 nM) + Lip-1 (500 nM) for 6 hours. After treatment, cells were washed with PBS and incubated with C11-BODIPY (1 µM) for 30 minutes at 37 °C before they were harvested by trypsinization. Subsequently, cells were resuspended in 400 µL of PBS supplemented with 2% FBS followed by analysis using a flow cytometer (FACS Canto II, BD Biosciences). For riboflavin deprivation experiments, cells were pre-incubated in riboflavin-deficient medium or medium supplemented with riboflavin (1 µM) for 72 hours before undergoing the treatments described above.
Instrument	FACS Canto II (BD Biosciences). Data was collected from the FITC detector (for the oxidized form of BODIPY) with a 502LP and 530/30 BP filter and from the PE detector (for the reduced form of BODIPY) with 556 LP and 585/42 BP filter. At least 10,000 events were analyzed per sample.
Software	For data collection the BD FACSDiva Software v6.1.3 was used.<br>For data analysis FlowJo v10.10.0 software was used.
Cell population abundance	The abundance of the desired cell population in post-sort fractions was generally > 85% of the total post-sort population. The ratio FITC/PE (oxidized/reduced ratio) was calculated as follows: (median FITC-A fluorescence−median FITC-A fluorescence of unstained samples)/(median PE-A fluorescence−median PE-A fluorescence of unstained samples).
Gating strategy	Live cell populations were separated from cellular debris and dead cells using SSC-A vs. FSC-A gating strategy.

☐ Tick this box to confirm that a figure exemplifying the gating strategy is provided in the Supplementary Information.
