# Supplementary figures and images for: Riboflavin metabolism shapes FSP1-driven ferroptosis resistance
Source: Nat Cell Biol. 2026 Mar 13;28(4):696–706. doi: 10.1038/s41556-025-01856-x (PMC13086581; doi:10.1038/s41556-025-01856-x)

Fig. 1b

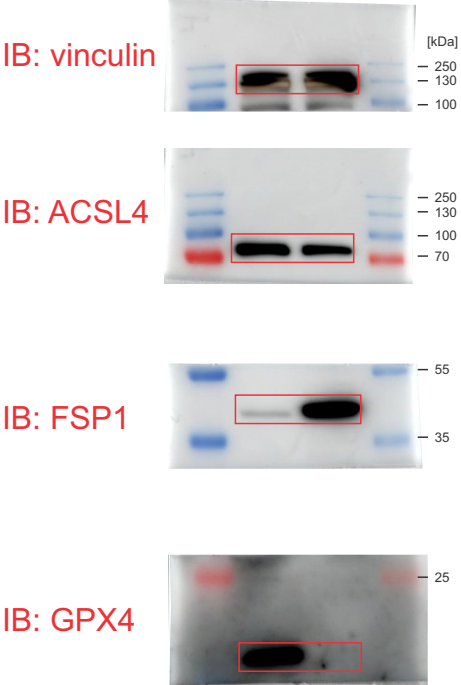

Fig. 1f

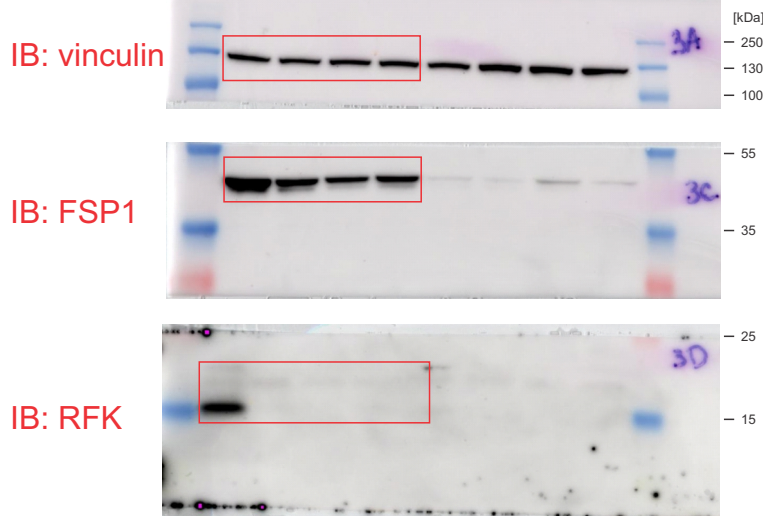

Supplement: Supplementary file 13 — Unprocessed western blots [file 41556_2025_1856_MOESM13_ESM.pdf]

Fig. 2c

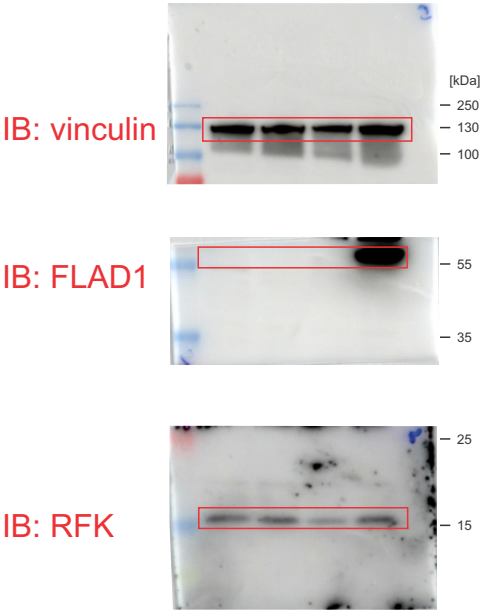

Fig. 2j

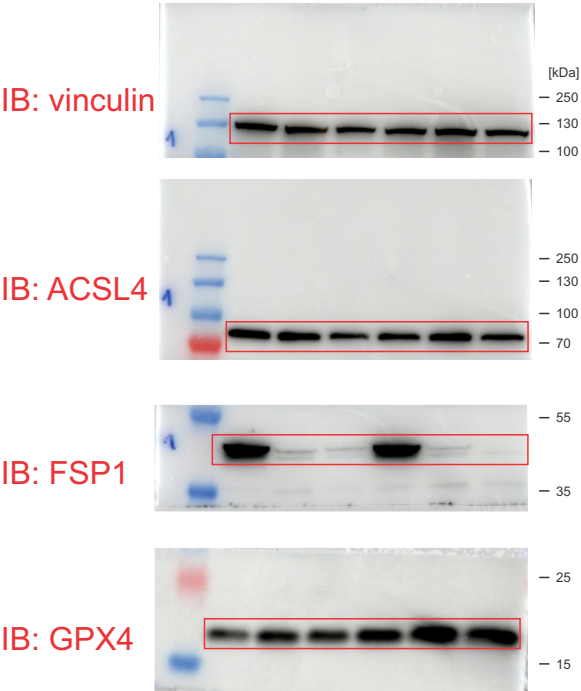

Supplement: Supplementary file 14 — Unprocessed western blots [file 41556_2025_1856_MOESM14_ESM.pdf]

Fig. 3b

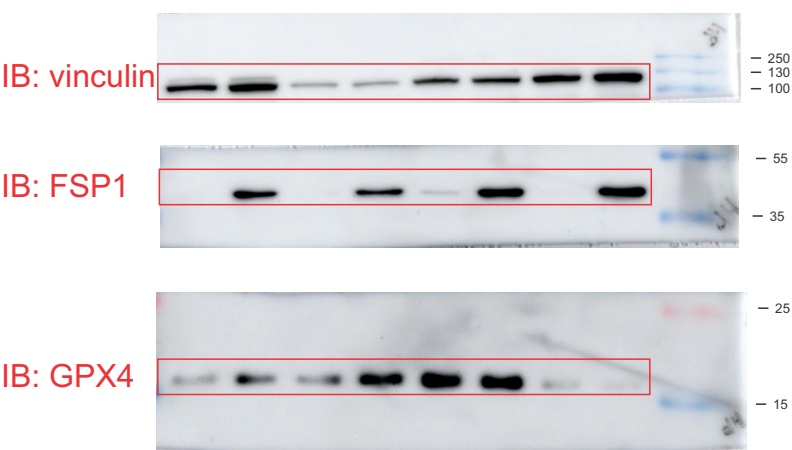

Fig. 3f

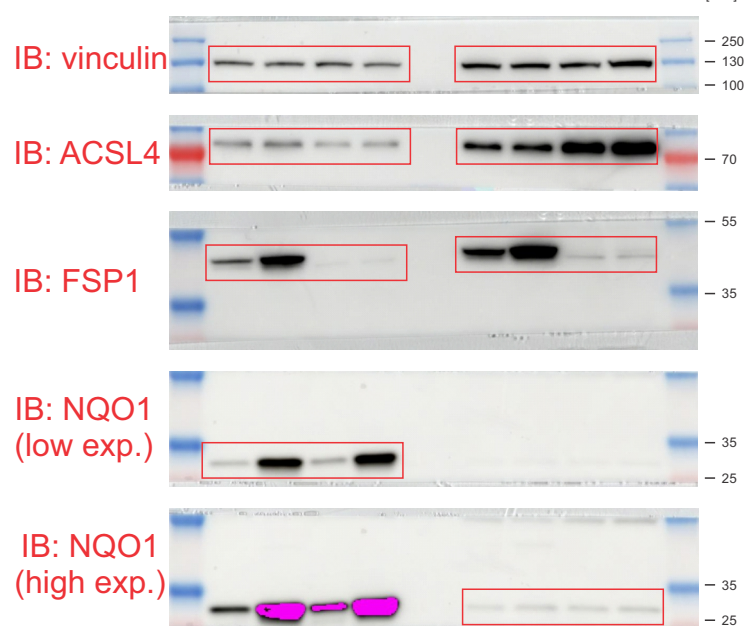

Supplement: Supplementary file 15 — Unprocessed western blots [file 41556_2025_1856_MOESM15_ESM.pdf]

Fig. 4d

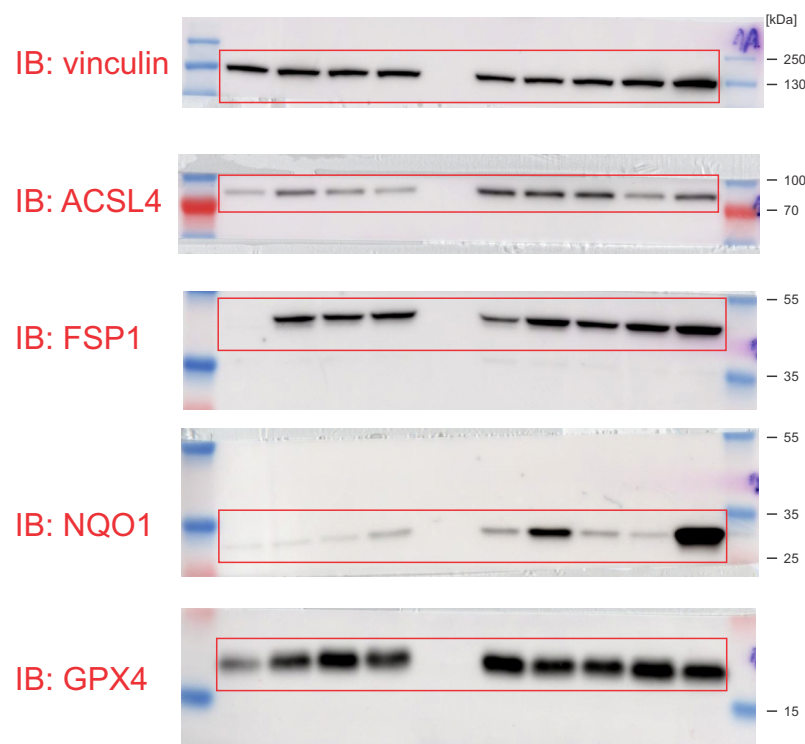

Fig. 4f

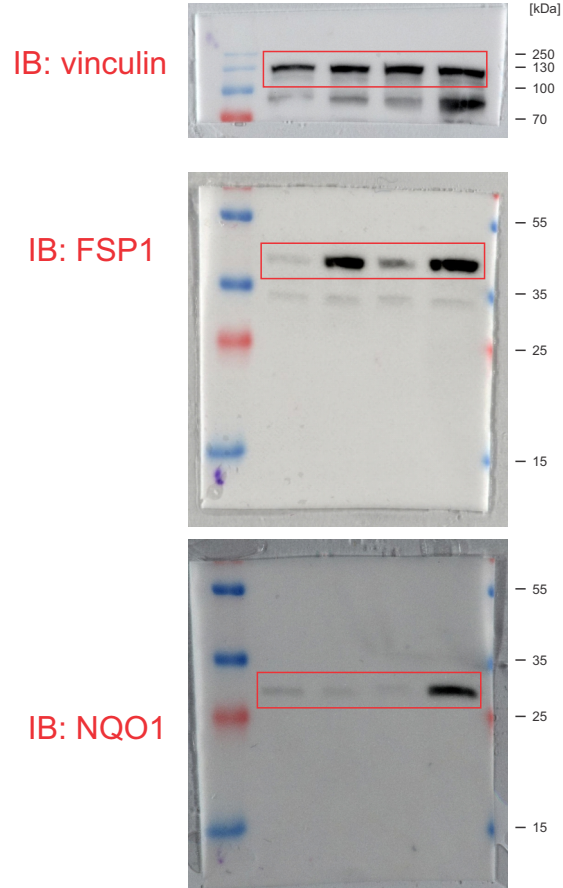

Fig. 4f

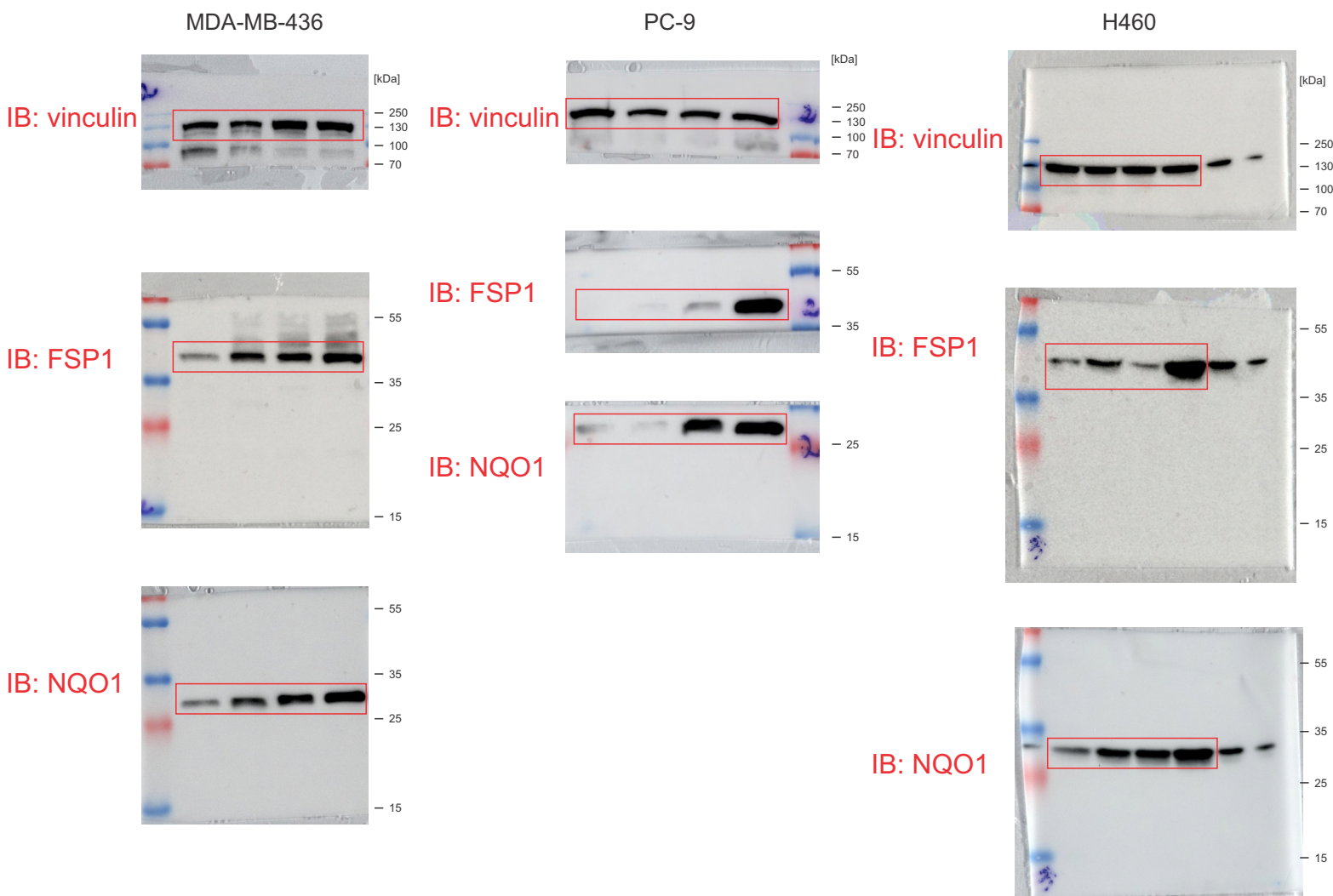

Supplement: Supplementary file 16 — Unprocessed western blots [file 41556_2025_1856_MOESM16_ESM.pdf]

Fig. 5a

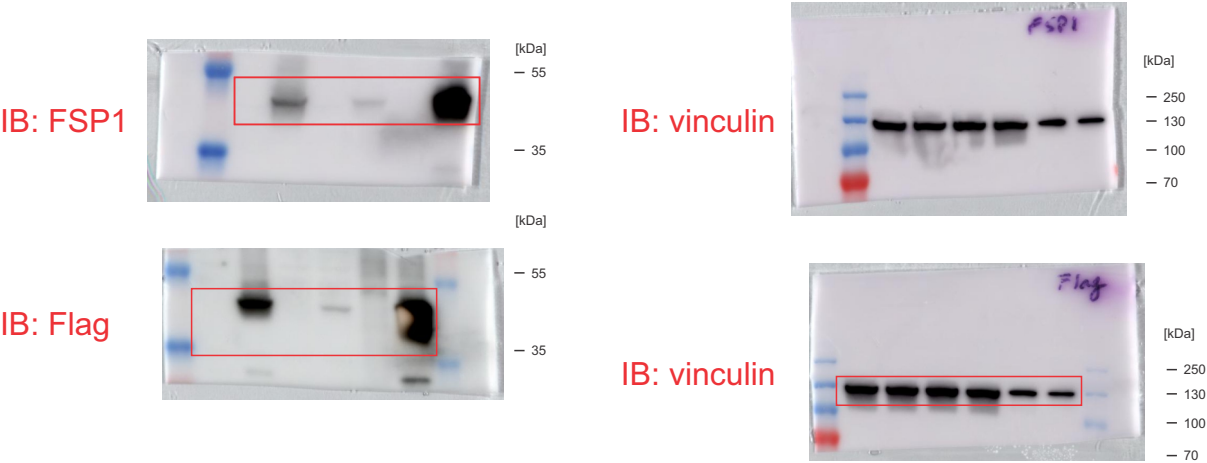

Fig. 5c

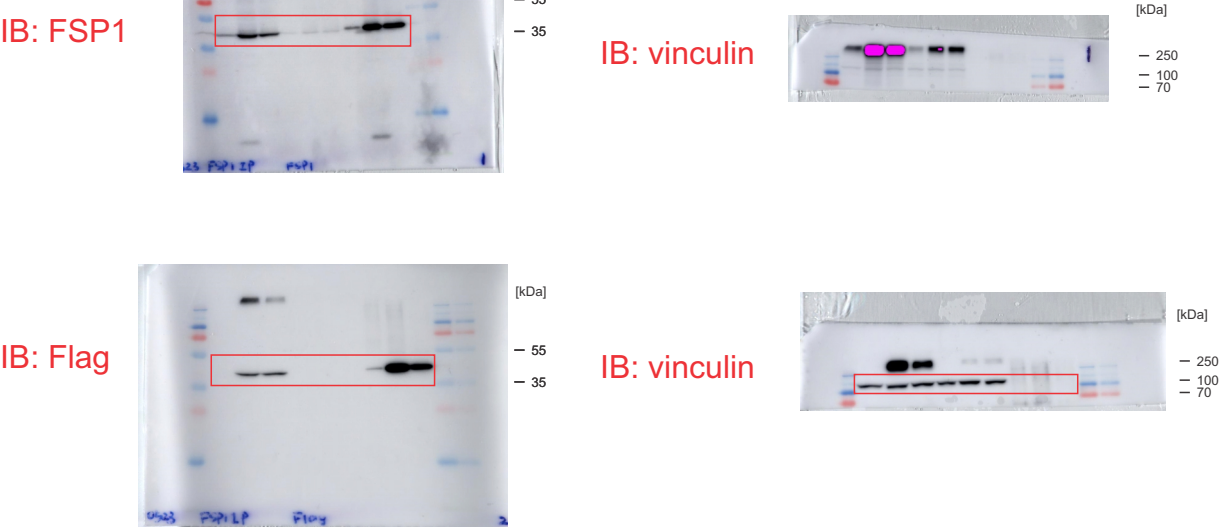

Supplement: Supplementary file 17 — Unprocessed western blots [file 41556_2025_1856_MOESM17_ESM.pdf]

Ex. Fig. 1e

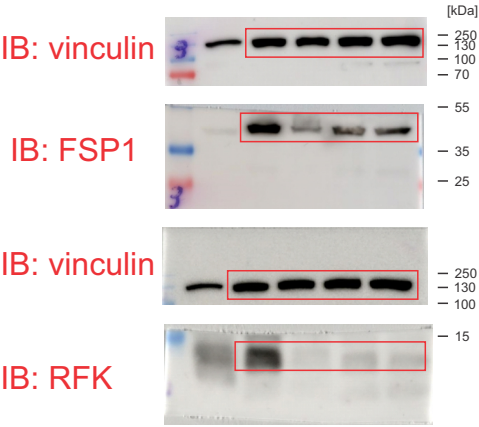

Supplement: Supplementary file 23 — Unprocessed western blots [file 41556_2025_1856_MOESM23_ESM.pdf]

Ex. Fig. 2a

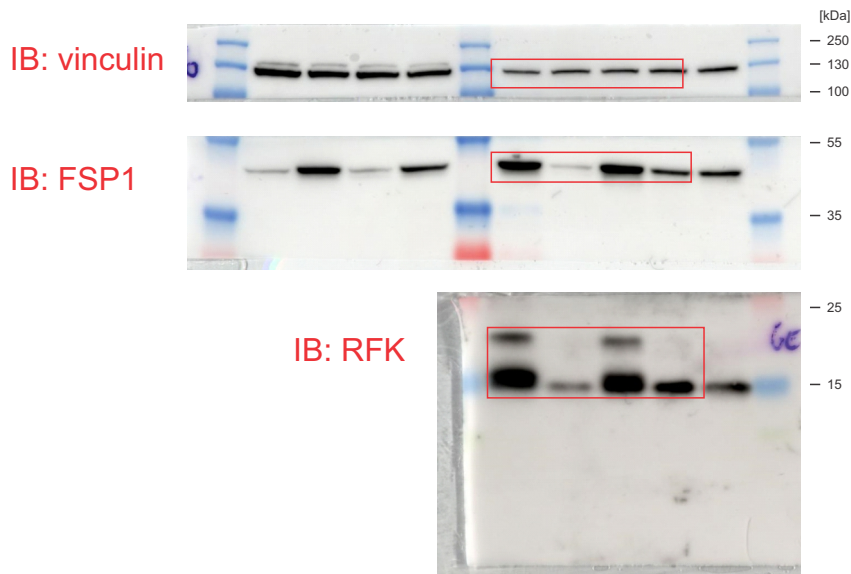

Ex. Fig. 2b

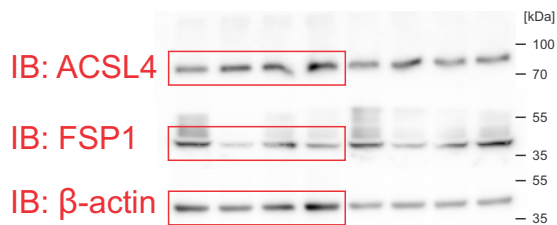

Ex. Fig. 2j

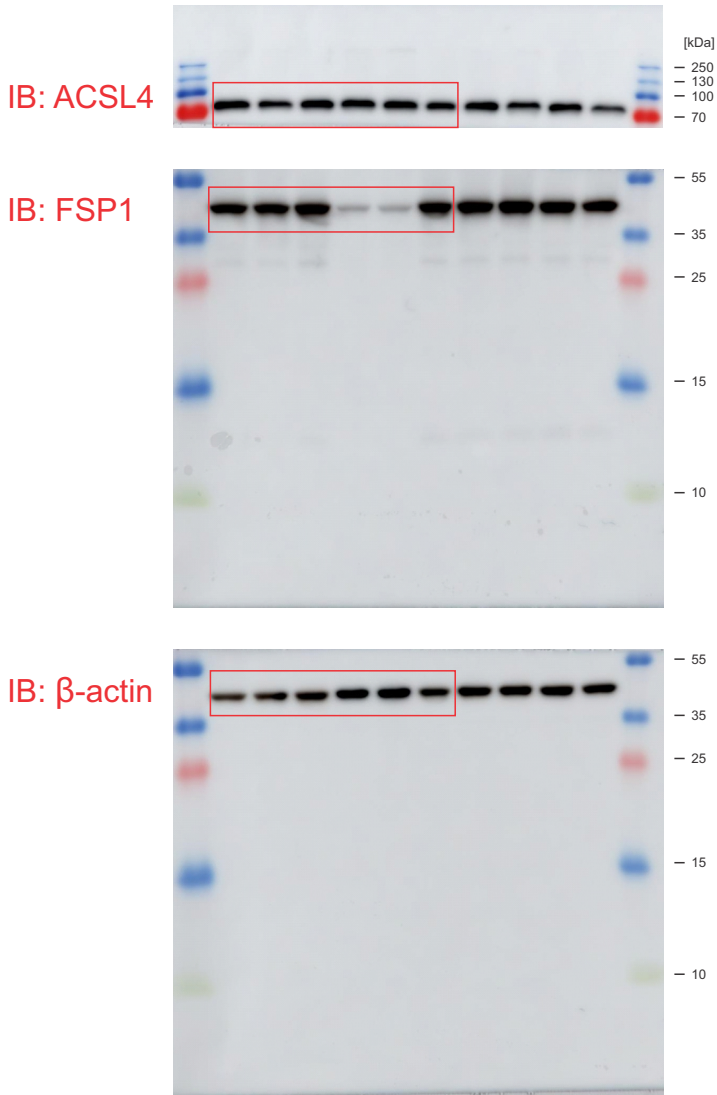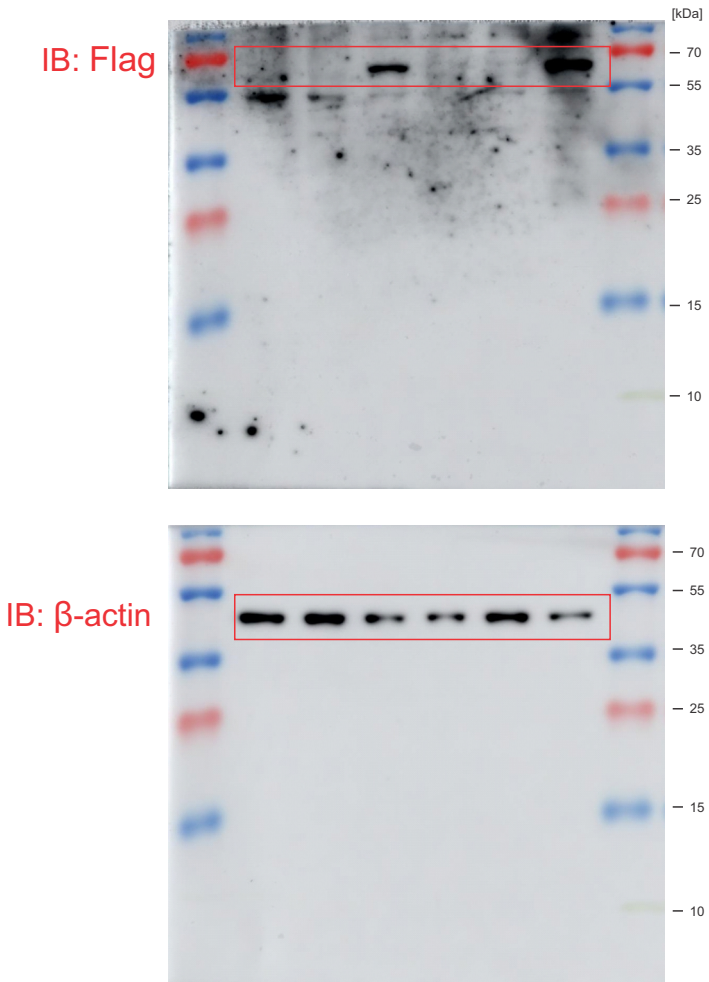

Ex. Fig. 2I

IB: vinculin

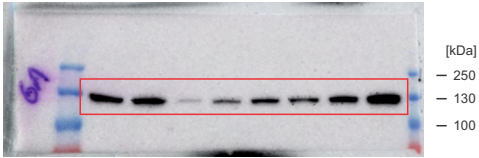

IB: FSP1

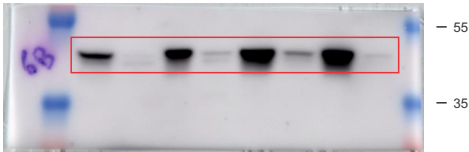

IB: GPX4

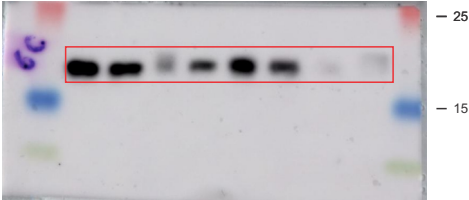

Supplement: Supplementary file 24 — Unprocessed western blots [file 41556_2025_1856_MOESM24_ESM.pdf]

Ex. Fig. 3a

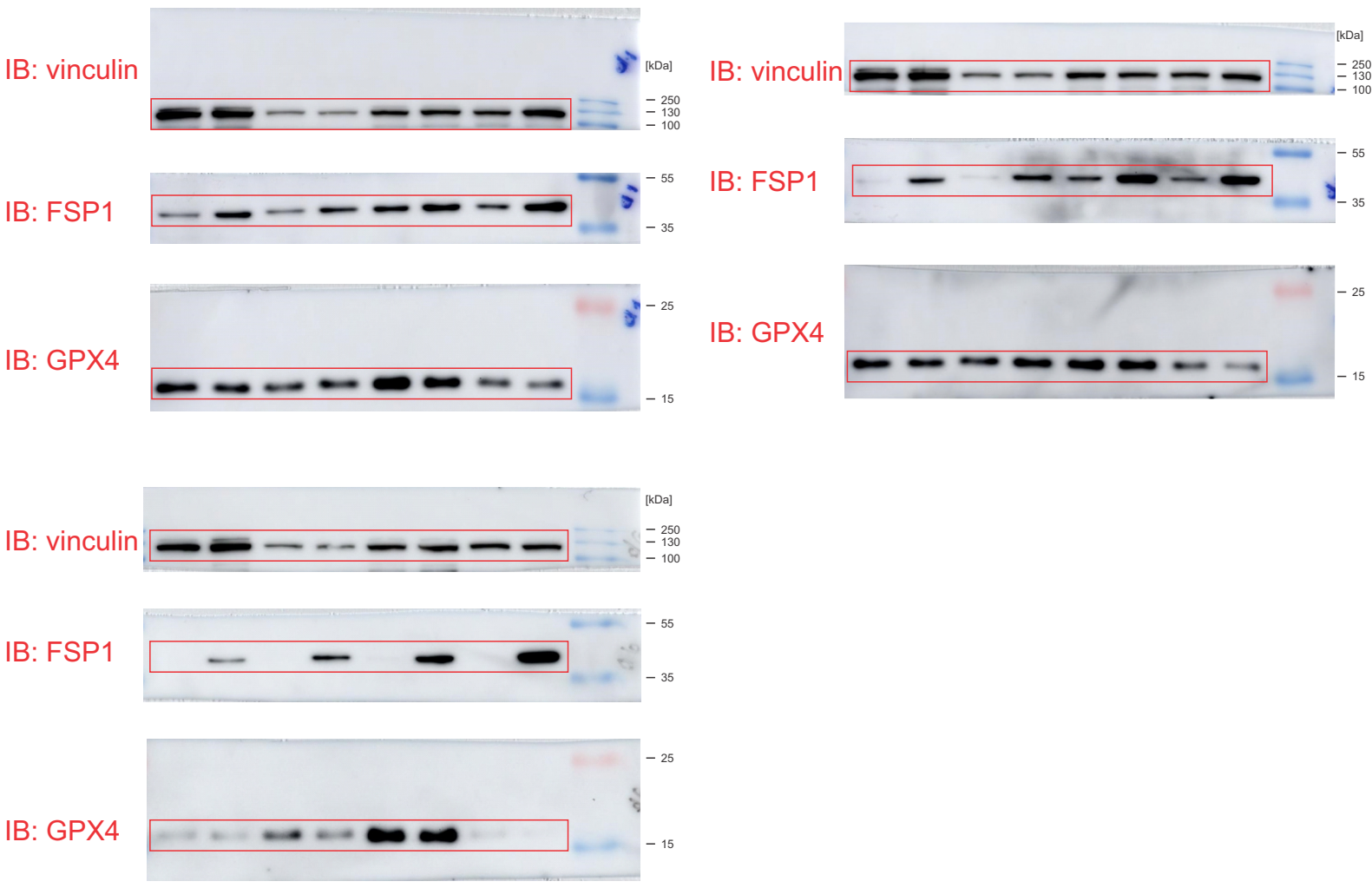

Supplement: Supplementary file 25 — Unprocessed western blots [file 41556_2025_1856_MOESM25_ESM.pdf]

Ex. Fig. 4c

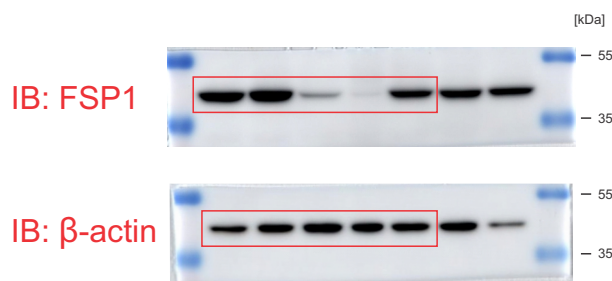

Ex. Fig. 4h

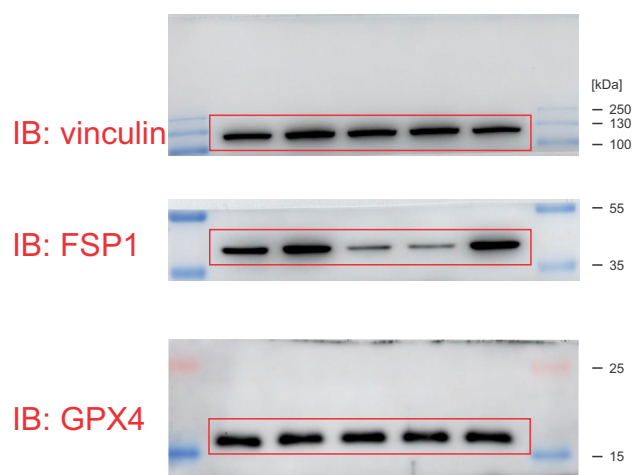

Ex. Fig. 4j

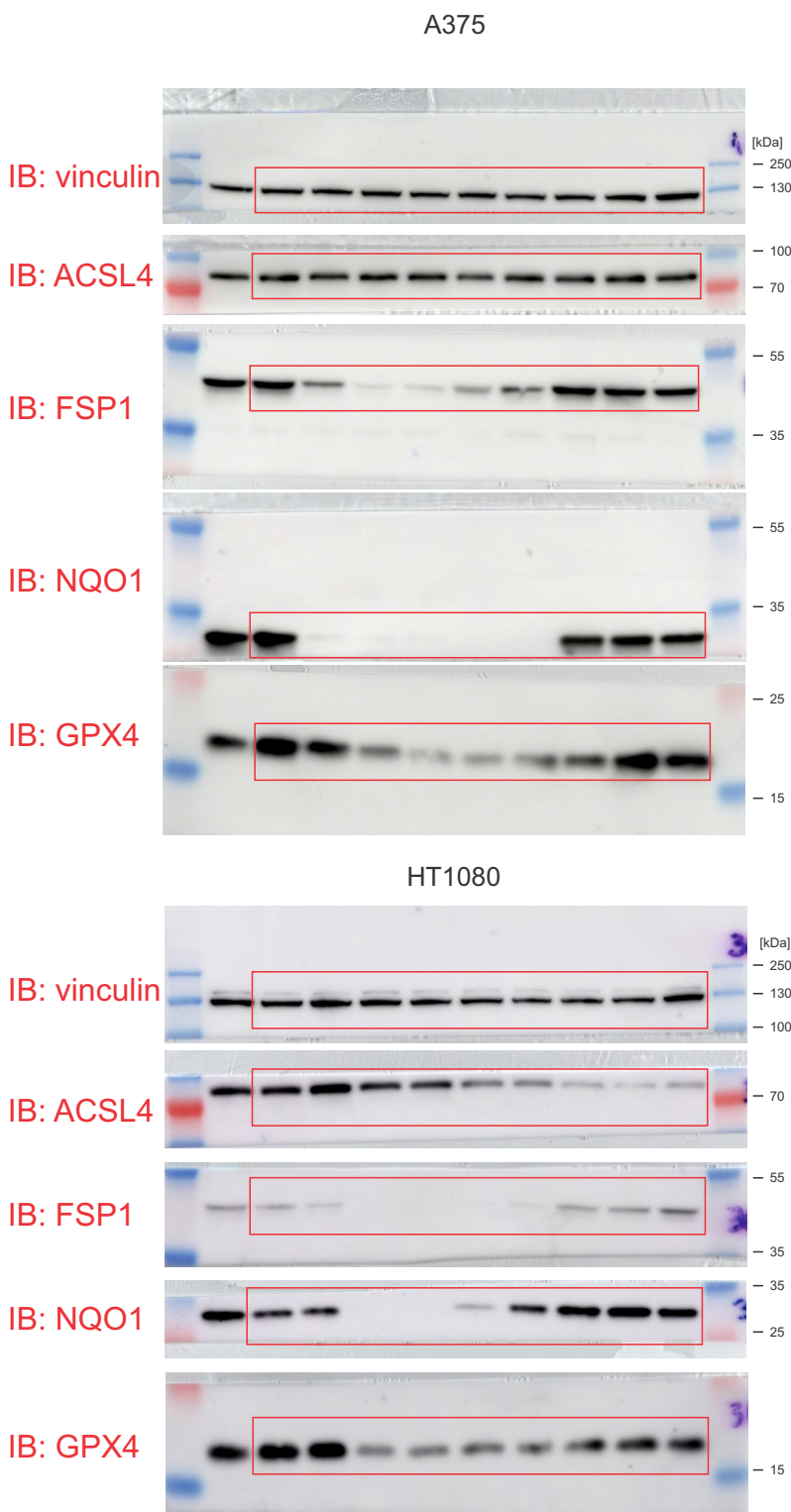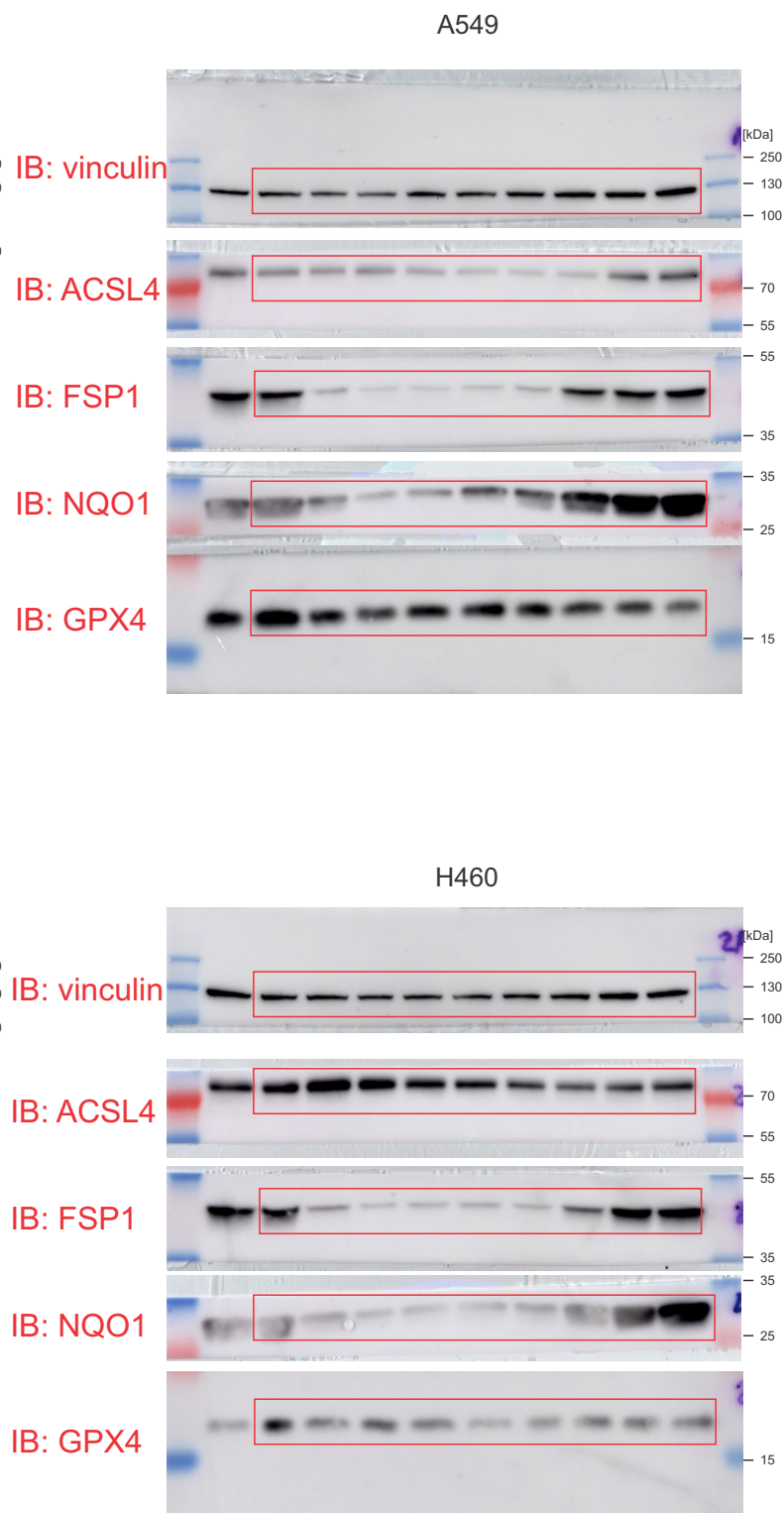

Supplement: Supplementary file 26 — Unprocessed western blots [file 41556_2025_1856_MOESM26_ESM.pdf]

Ex. Fig. 5c

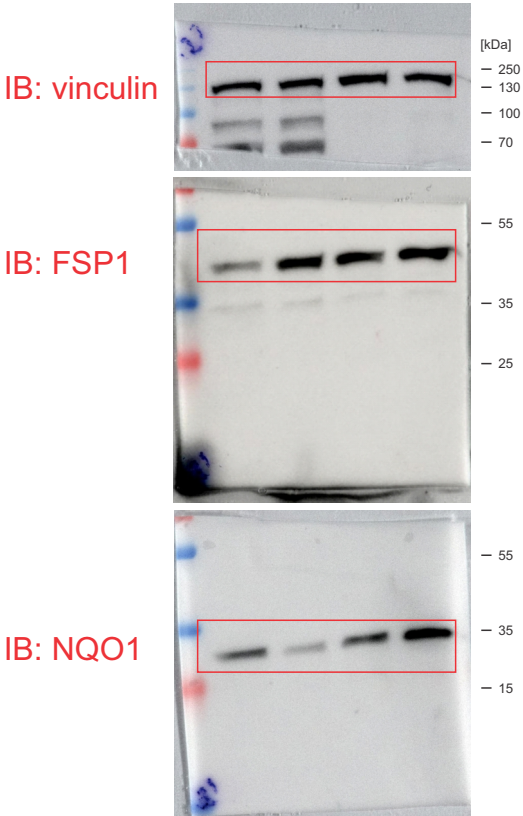

Supplement: Supplementary file 27 — Unprocessed western blots [file 41556_2025_1856_MOESM27_ESM.pdf]
